# Counseling Received by Adolescents Undergoing Voluntary Medical Male Circumcision: Moving Toward Age-Equitable Comprehensive Human Immunodeficiency Virus Prevention Measures

**DOI:** 10.1093/cid/cix952

**Published:** 2018-04-03

**Authors:** Michelle R Kaufman, Eshan U Patel, Kim H Dam, Zoe R Packman, Lynn M Van Lith, Karin Hatzold, Arik V Marcell, Webster Mavhu, Catherine Kahabuka, Lusanda Mahlasela, Emmanuel Njeuhmeli, Kim Seifert Ahanda, Getrude Ncube, Gissenge Lija, Collen Bonnecwe, Aaron A R Tobian

**Affiliations:** 1 Johns Hopkins Bloomberg School of Public Health, Baltimore, Maryland; 2 Department of Pathology, Johns Hopkins University School of Medicine, Baltimore, Maryland; 3 Johns Hopkins Center for Communication Programs, Baltimore, Maryland; 4 Population Services International, Harare, Zimbabwe; 5 Department of Pediatrics, Johns Hopkins University School of Medicine, Baltimore, Maryland; 6 Centre for Sexual Health & HIV/AIDS Research, Harare, Zimbabwe; 7 CSK Research Solutions, Ltd., Dar es Salaam, Tanzania; 8 Centre for Communication Impact, Pretoria, South Africa; 9 Office of HIV/AIDS, Global Health Bureau, United States Agency for International Development, Washington, District of Columbia; 10 Ministry of Health and Child Care, Harare, Zimbabwe; 11 Ministry of Health, Community Development, Gender, Elderly and Children, Dar es Salaam, Tanzania; 12 National Department of Health, Pretoria, South Africa

**Keywords:** adolescents, voluntary medical male circumcision (VMMC), HIV prevention, HIV counseling, sub-Saharan Africa

## Abstract

**Background:**

The minimum package of voluntary medical male circumcision (VMMC) services, as defined by the World Health Organization, includes human immunodeficiency virus (HIV) testing, HIV prevention counseling, screening/treatment for sexually transmitted infections, condom promotion, and the VMMC procedure. The current study aimed to assess whether adolescents received these key elements.

**Methods:**

Quantitative surveys were conducted among male adolescents aged 10–19 years (n = 1293) seeking VMMC in South Africa, Tanzania, and Zimbabwe. We used a summative index score of 8 self-reported binary items to measure receipt of important elements of the World Health Organization–recommended HIV minimum package and the US President’s Emergency Plan for AIDS Relief VMMC recommendations. Counseling sessions were observed for a subset of adolescents (n = 44). To evaluate factors associated with counseling content, we used Poisson regression models with generalized estimating equations and robust variance estimation.

**Results:**

Although counseling included VMMC benefits, little attention was paid to risks, including how to identify complications, what to do if they arise, and why avoiding sex and masturbation could prevent complications. Overall, older adolescents (aged 15–19 years) reported receiving more items in the recommended minimum package than younger adolescents (aged 10–14 years; adjusted β, 0.17; 95% confidence interval [CI], .12–.21; *P* < .001). Older adolescents were also more likely to report receiving HIV test education and promotion (42.7% vs 29.5%; adjusted prevalence ratio [aPR], 1.53; 95% CI, 1.16–2.02) and a condom demonstration with condoms to take home (16.8% vs 4.4%; aPR, 2.44; 95% CI, 1.30–4.58). No significant age differences appeared in reports of explanations of VMMC risks and benefits or uptake of HIV testing. These self-reported findings were confirmed during counseling observations.

**Conclusions:**

Moving toward age-equitable HIV prevention services during adolescent VMMC likely requires standardizing counseling content, as there are significant age differences in HIV prevention content received by adolescents.

Male circumcision reduces human immunodeficiency virus (HIV) acquisition [1–5], and voluntary medical male circumcision (VMMC) services offer a prime opportunity to promote comprehensive prevention measures, including HIV testing and other sexual health interventions. The World Health Organization (WHO) recommends integrating the standard WHO HIV prevention minimum package with the VMMC platform, such that all patients undergoing VMMC receive an age-appropriate combination of a minimum package of services, including but not limited to (1) an offer of HIV testing and counseling, (2) screening and treatment for sexually transmitted infections, (3) promotion and provision of condoms, (4) promotion of safer sex practices and risk reduction counseling, and (5) the VMMC procedure [6]. Although the US President’s Emergency Plan for AIDS Relief (PEPFAR) has released a list of best practices for VMMC site operations [7], it is unknown whether these recommendations are followed consistently for boys and men of all age groups, particularly adolescents, across all VMMC priority countries. It is also unknown whether the content and quality of counseling sessions are age appropriate and adapted to adolescent needs.

Recently, a qualitative study suggested that information on HIV prevention was not being provided as thoroughly to younger adolescents as older adolescents [8]. In this multicountry study, we aimed to assess whether key elements of WHO’s recommended minimum package were received by adolescents seeking VMMC and to identify what counseling content is being delivered, what content is missing, and whether content varies by adolescent age group.

## METHODS

### Ethics Statement

The Human Sciences Research Council in South Africa, Tanzania National Institute for Medical Research, Medical Research Council of Zimbabwe, and Johns Hopkins Bloomberg School of Public Health Institutional Review Board approved the study before data collection.

### Settings, Participants, and Procedures

Data were collected from June 2015 to September 2016 in South Africa, Tanzania, and Zimbabwe from 14 VMMC sites (4 in South Africa, 4 in Tanzania, and 6 in Zimbabwe), as described elsewhere [9, 10].

#### Adolescent Surveys

Surveys were conducted with a convenience sample of male adolescents (aged 10–19 years) seeking VMMC, who were recruited by VMMC providers and/or community mobilizers working with trained coordinators. Adolescents provided consent if aged 18 to 19 years or assent and parent permission if a minor. Initial surveys were conducted before adolescents received any VMMC counseling or underwent the procedure. A follow-up survey was conducted approximately 7–10 days after the procedure, during the adolescent’s follow-up clinic appointment or in his home if he did not attend the appointment. Trained data collectors administered the surveys in the language of the participants’ choice (Sesotho, isiZulu, or isiSwati in South Africa; kiSwahili in Tanzania; Shona or Ndebele in Zimbabwe). Of the 1526 participants who underwent VMMC, 233 (15.3%) did not complete the follow-up survey. Data from participants who completed both the initial and follow-up surveys were included in the primary analysis of this study (South Africa, n = 299; Tanzania, n = 498; Zimbabwe, n = 496).

#### Counseling Observations

A subset of selected survey participants consented to recording their counseling sessions for observation. Counseling sessions were video and audio recorded (for individual and group counseling sessions) in Tanzania (n = 20); video recorded (for individual sessions) in Zimbabwe (n = 20), and audio recorded only (for individual sessions) in South Africa (n = 4). Observations were not linked with surveys for privacy reasons. Although VMMC programs in each country are recommended to conduct group, individual, and postoperative counseling, not all countries implemented all of these components; therefore, observations could not be made for all formats in each country. Informed consent was obtained from providers whose counseling sessions were recorded. A local trained data collector used a counseling observation checklist to note the presence or absence of elements in the session, the quality of the counseling interaction, and the tone of the counselor. Procedures regarding counseling observations are further detailed in the Supplemental Material.

### Measures: Individual Surveys

#### World Health Organization-Recommended Human Immunodeficiency Virus Minimum Package Index

We measured receipt of a subset of important elements of the WHO-recommended HIV minimum package and PEPFAR VMMC recommendations for adolescents [7], using a summative overall index score created for this study of 8 self-reported binary items. The overall index score was subdivided into 4 sections: 3 subindices and 1 consent/assent proxy. The VMMC subindex reflects explanation of VMMC risks and benefits; the HIV test promotion subindex reflects the offer of HIV testing, encouragement for testing, and an explanation of the importance of testing; and the condom subindex includes condoms to take home and a demonstration of how to properly use a condom. The consent/assent proxy was based on two survey items that asked if participants were offered HIV testing and if participants received an explanation that it was their choice whether or not to be tested for HIV before VMMC.

#### Receipt of Human Immunodeficiency Virus Testing

Participants were asked, “Before the procedure, were you tested for HIV?” Binary responses were coded as 0 (no/don’t remember) or 1 (yes).

### Measures: Counseling Observations

The observed counseling sessions assessed the patient-provider interaction, including communication style, age-appropriate speech, and a tone of attention and respect toward the adolescent. The observation checklist was adapted from previously designed tools to assess health services for adolescents [11, 12]. Counseling elements were coded as 0 (not observed) or 1 (observed).

### Statistical Analysis

For the primary analysis, we examined correlates of the overall index score for counseling content (0–8; continuous outcome). Because the primary independent variable of interest was age group, we next examined age-group associations for each complete subindex (VMMC subindex [2/2], HIV subindex [2/2], condom subindex [2/2], and consent/assent proxy [2/2]) and receipt of HIV testing as binary outcomes. Complete subindices were analyzed instead of each item separately, as they better reflect current standards and completeness of the content and services adolescents should be receiving. These analyses used Poisson regression models with generalized estimating equations and robust variance estimation to allow for clustering at the facility level. Hypothesized confounding factors and those shown to have an association with the outcome in univariable models were included in the final multivariable models.

Because the primary analysis included only adolescents who completed a postprocedure survey, we conducted a sensitivity analysis using multiple imputation by chained equations (MICE) to account for potential selection bias among participants who underwent VMMC but did not complete a postprocedure survey (n = 233/1526) [9]. Predictors were selected based on unadjusted correlations with outcome variables. Highly collinear predictors were dropped. Twenty imputations were conducted.

Descriptive proportions for counseling observations were calculated, and differences between age groups were assessed using Fisher’s exact test for exploratory purposes. All analyses were performed using State SE software, version 14.2 (StataCorp).

## RESULTS

### Sociodemographics

Table 1 shows characteristics of the survey sample by age group (10–14 or 15–19 years) and overall. A majority of the adolescent participants were from urban areas (53.8%). A majority of older adolescents reported receiving individual counseling only (42.2%), whereas a majority of younger adolescents reported receiving both individual and group counseling (48.8%). A parent/guardian was more likely to attend preprocedure counseling with younger adolescents than with older adolescents (56.1% vs 12.5%, respectively). The vast majority of younger adolescents reported no sexual experience (94.1%), whereas a little more than a third of the older adolescents reported some sort of sexual experience (37.4%).

**Table 1.** Sample Characteristics

Characteristic	Adolescents, No. (%)		
	Aged 10–14 y (n = 836)	Aged 15–19 y (n = 457)	Overall (n = 1293)
Country			
South Africa	187 (22.4)	112 (24.5)	299 (23.1)
Tanzania	413 (49.4)	85 (18.6)	498 (38.5)
Zimbabwe	236 (28.2)	260 (56.9)	496 (38.4)
Facility setting			
Urban	429 (51.3)	267 (58.4)	696 (53.8)
Periurban	149 (17.8)	43 (9.4)	192 (14.8)
Rural	258 (30.9)	147 (32.2)	405 (31.3)
Preprocedure counseling			
Individual only	139 (16.6)	193 (42.2)	332 (25.7)
Group only	282 (33.7)	175 (38.3)	457 (35.3)
Both	408 (48.8)	87 (19.0)	495 (38.3)
Parent/guardian attendance at preprocedure counseling			
No	361 (43.2)	399 (87.3)	760 (58.8)
Yes	469 (56.1)	57 (12.5)	526 (40.7)
Received postprocedure counseling session^a^			
No	656 (78.5)	233 (51.0)	889 (68.8)
Yes	180 (21.5)	224 (49.0)	404 (31.2)
Ever had sexual experience			
No	787 (94.1)	286 (62.6)	1073 (83.0)
Yes	48 (5.7)	171 (37.4)	219 (16.9)

^a^All participants received pre-procedure counseling. Some participants received an additional postprocedure counseling session. Percentages may not add up to 100 due to missing data.

### Counseling Content as Reported by Adolescents

Table 2 shows the percentage of adolescents who reported receiving each item included in the index, disaggregated by younger (10–14 years) and older (15–19 years) age. Table 3 shows receipt of complete subindices by age group. In the multivariable model accounting for country, counseling type (group and/or individual), having ever had a sexual encounter, and receipt of a postprocedure counseling session, 15–19-year-olds were more likely than younger adolescents to report receiving all elements of the HIV test promotion subindex (42.7% vs 29.5%; adjusted prevalence ratio [aPR], 1.53; 95% confidence interval [CI], 1.16–2.02). In addition, 15–19-year-olds were significantly more likely than 10–14-year-olds to report receiving both a condom demonstration and condoms to take home (condom subindex; 16.8% vs 4.4%; aPR, 2.44; 95% CI, 1.30–4.58).

**Table 2.** Reported Receipt of Items in the World Health Organization-Recommended Human Immunodeficiency Virus Minimum Package Index

Index Item	Interview Question(s)	Adolescents, % (No./Total No.)^a^		
		Aged 10–14 y	Aged 15–19 y	Overall
VMMC subindex (range, 0–2)				
Explanation of VMMC benefits	Did the person who counseled/educated you explain to you why it was good to get circumcised?	83.1 (694/835)	91.2 (415/455)	86.0 (1109/1209)
Explanation of VMMC risks	Did the person who counseled/educated you explain to you the risks of getting circumcised?	51.7 (432/835)	63.7 (290/455)	56.0 (722/1290)
HIV test promotion subindex (range, 0–2)				
Explanation of HIV test importance	Were you told why it was important to have an HIV test before being circumcised?	34.7 (290/835)	47.5 (217/457)	39.2 (507/1292)
Encouragement for HIV testing	Did the person who counseled/educated you encourage you to be tested for HIV?	70.3 (586/834)	82.8 (376/454)	74.7 (962/1288)
Condom subindex (range, 0–2)				
Received condoms to take home	Did the person who counseled/educated you give you condoms to take home?	4.7 (37/781)	19.0 (86/452)	10.0 (123/1233)
Received a demonstration on how to use a condom^b^	Did the person who counseled/educated you show you how to use a condom properly (gave a demonstration)? *or* Did the counselor show you how to use a condom?	17.9 (150/836)	51.6 (236/457)	29.9 (386/1293)
Consent/assent proxy (0–2)				
Offer of HIV Testing	Before the circumcision, were you asked if you wanted to have an HIV test?	67.4 (563/835)	79.0 (360/457)	71.4 (923/1292)
Explanation that HIV testing was the patient’s choice	Did the person who counseled/educated you explain that it was your choice whether or not to be tested for HIV before you were circumcised?	49.2 (410/833)	61.9 (281/454)	53.7 (691/1287)
Overall index score				
Median (interquartile range)		4 (2–5)	5 (4–7)	4 (3–6)
Mean (standard deviation)		3.8 (1.87)	5.0 (1.94)	4.2 (1.98)

Abbreviations: HIV, human immunodeficiency virus; VMMC, voluntary male medical circumcision.

^a^Data represent % (No./Total No.) of adolescents unless otherwise specified.

^b^“Received a demonstration how to use a condom” is a composite variable. Answers were considered positive if respondent answered yes to either “Did the person who counseled/educated you show you how to use a condom properly (gave you a demonstration)?” or “Did the counselor show you how to use a condom?”

**Table 3.** Receipt of Complete Subindices and HIV Testing Uptake by Age Group^a^

Subindex	Age Group, y	Adolescents, % (No./Total No.)	PR (95% CI)	aPR^b^ (95% CI)
Complete VMMC subindex	10–14	48.0 (401/836)	Reference	Reference
	15–19	61.3 (280/457)	1.15 (1.03–1.28)^c^	1.06 (0.95–1.19)
Complete HIV prevention subindex	10–14	29.5 (247/836)	Reference	Reference
	15–19	42.7 (195/457)	1.53 (1.19–1.97)^c^	1.53 (1.16–2.02)^c^
Complete condom subindex	10–14	4.4 (37/836)	Reference	Reference
	15–19	16.8 (77/457)	2.76 (1.47–5.19)^c^	2.44 (1.30–4.58)^c^
Complete consent/assent (proxy)	10–14	39.0 (326/836)	Reference	Reference
	15–19	53.6 (245/457)	1.18 (1.04–1.33)^c^	1.19 (1.01–1.40)^c^
Receipt of HIV testing	10–14	88.0 (736/836)	Reference	Reference
	15–19	94.3 (431/457)	1.02 (0.96–1.08)	1.02 (0.97–1.08)

Abbreviations: aPR, adjusted prevalence ratio; CI, confidence interval; HIV, human immunodeficiency virus; PR, prevalence ratio; VMMC, voluntary medical male circumcision.

^a^The outcome was a complete subindex (VMMC subindex, 2/2; HIV subindex, 2/2; condom subindex, 2/2; consent proxy, 2/2). PRs were calculated by modified Poisson regression models with generalized estimating equations and robust variance estimators to account for clustering of responses at the facility level.

^b^Final multivariable models for each subindex included adjustment for country, preprocedure counseling mode, ever having had a sexual encounter, and receipt of a postprocedure counseling session.

^c^ *P* value < .05.

Nearly all adolescents reported having an HIV test before the VMMC procedure (88.0% aged 10–14 and 94.3% aged 15–19 years; Table 3). There was no significant difference by age group for receipt/uptake of HIV testing (aPR, 1.02; 95% CI, .97–1.08). Furthermore, all reported associations between age and each subindex and HIV testing uptake were nearly identical when using the imputed data set (Supplementary Table S1).

### Factors Associated With Overall Index Score

Table 4 shows factors associated with higher overall index scores for elements adolescents reported receiving during counseling. Older age group (15–19 years), the presence of a postprocedure counseling session, and having ever had any sexual experience were all significantly associated with a higher overall index score. On the other hand, receiving group counseling only was associated with lower overall index scores in the univariable model. In the final multivariable model, older age group (15–19 years), and the presence of a postprocedure counseling session remained independently associated with higher overall index scores.

**Table 4.** Factors Associated With Higher Overall Index Scores for Counseling Content

Characteristic	β^a^ (95% CI)	aβ^b^ (95% CI)
Age Group, y		
10–14	Reference	Reference
15–19	0.20 (.16, .24)^c^	0.17 (.12, .21)^c^
Facility setting		
Urban	Reference	…
Periurban	0.07 (−.21, .35)	…
Rural	0.15 (−.07, .36)	…
Preprocedure counseling mode		
Individual only	Reference	Reference
Group only	−0.13 (−.20, −.06)^c^	−0.10 (−.17, −.04)^c^
Both	−0.11 (−.20, −.01)^c^	−0.05 (−.15, .05)
Parent/guardian attendance at preprocedure counseling session		
No	Reference	…
Yes	−0.09 (−.18, .00)	…
Postprocedure counseling		
No	Reference	Reference
Yes	0.20 (.14, .26)^c^	0.16 (.10, .23)^c^
Ever had any sexual experience		
No	Reference	Reference
Yes	0.13 (.07, .18)^c^	0.02 (−.06, .10)

Abbreviations: CI, confidence interval.

^a^βs and 95% CIs were calculated by modified Poisson regression models with generalized estimation equations and robust variance estimators to account for clustering of responses at the facility level.

^b^Final multivariable model included adjustment for country, facility setting, preprocedure counseling mode, ever having had a sexual encounter, and receipt of postprocedure counseling session.

^c^ *P* < .05.

### Counseling Session Observations

Counseling observations (n = 44; Table 5) revealed that few adolescents were asked if they had ever had sex (16.7% aged 10–14 and 30.4% aged 15–19 years) or were sexually active (16.7% and 26.1%, respectively). Although providers were consistent in explaining the benefits of VMMC (observed in all sessions) and the meaning of partial protection from HIV after the procedure (explained to 88.9% aged 10–14 and 95.7% aged 15–19 years), only two-thirds of the sessions included an explanation of the procedure risks. Providers also did not consistently encourage adolescents to be tested for HIV but offered them the option to decline and continue with the procedure (item most similar to consent/assent proxy, described above; 77.8% for adolescents aged 10–14 and 60.9% for those aged 15–19 years). About a quarter of counselors observed did not tell the adolescent that his HIV test results would be shared with him (information provided to 77.8% aged 10–14 and 78.3% aged 15–19 years).

**Table 5.** Counseling Session Observations

Sample Observation Items	Adolescents, No. (%)		
	Overall (n = 44)^a^	Aged 10–14 y (n = 18)	Aged 15–19 y (n = 23)
**Provider introduction**			
Greets the adolescent	41 (93.2)	16 (88.9)	22 (95.7)
Tells the adolescent his/her name	41 (93.2)	15 (83.3)	23 (100.0)
Asks for the adolescent’s name	32 (72.7)	16 (88.9)	14 (60.9)
Tells the adolescent he/she is going to ask some personal questions	13 (29.5)	1 (5.6)	11 (47.8)
Reassures the adolescent all information provided will be kept confidential	10 (22.7)	2 (11.1)	7 (30.4)
Ensures privacy in the counseling area	26 (59.1)	11 (61.1)	14 (60.9)
**Provider provided VMMC and HIV testing explanation**			
The benefits of VMMC	44 (100.0)	18 (100.0)	23 (100.0)
The risks associated with the procedure	28 (63.6)	12 (66.7)	15 (65.2)
The meaning of partial protection from HIV infection after VMMC	41 (93.2)	16 (88.9)	22 (95.7)
The importance of HIV testing	35 (79.5)	13 (72.2)	19 (82.6)
The adolescent is strongly urged to be tested for HIV but he may decline and still undergo the procedure	29 (65.9)	14 (77.8)	14 (60.9)
The HIV test result will be shared with the adolescent	35 (79.5)	14 (77.8)	18 (78.3)
**Provider provided postprocedure care explanation**			
How much pain the adolescent could experience during the procedure	38 (86.4)	17 (94.4)	20 (87.0)
How much pain the adolescent could experience after the procedure	36 (81.8)	16 (88.9)	19 (82.6)
How to take care of the wound	40 (90.9)	18 (100.0)	21 (91.3)
Why follow-up visits are needed	41 (93.2)	18 (100.0)	20 (87.0)
How to look for specific complications (infection, bleeding, etc)	29 (65.9)	12 (66.7)	15 (65.2)
What to do if complications arise	33 (75.0)	13 (72.2)	18 (78.3)
The importance of abstaining from sex for at least 6 wk after the procedure	19 (43.2)	5 (27.8)	13 (56.5)
The importance of abstaining from masturbation/self-sex for at least 6 wk	16 (36.4)	4 (22.2)	11 (47.8)
The need to use condoms even after being circumcised	23 (52.3)	7 (38.9)	15 (65.2)
How to use condoms (ie, condom demonstration)	6 (13.6)	0	5 (21.7)
The need to reduce the number of sexual partners even after circumcision	14 (31.8)	1 (5.6)	12 (52.2)
**Provider-adolescent communication**			
Asks questions to make sure the adolescent understands the information provided	44 (100.0)	18 (100.0)	23 (100.0)
Asks the adolescent to ‘teach back’ what he just learned to the provider	27 (61.4)	13 (72.2)	13 (56.5)
**Provider response to adolescent feedback**			
Asks the adolescent if he has (any other) questions or needs clarification	41 (93.2)	18 (100.0)	21 (91.3)
Allows the adolescent enough time to ask anything he wants	41 (93.2)	18 (100.0)	21 (91.3)
Answers the adolescent’s questions in a relaxed manner without seeming rushed	32 (72.7)	11 (61.1)	19 (82.6)
**Provider uses age-appropriate speech**			
Avoids the use of medical jargon	39 (88.6)	16 (88.9)	20 (87.0)
Asks if the adolescent has ever had sex	10 (22.7)	3 (16.7)	7 (30.4)
Asks if the adolescent is sexually active	9 (20.5)	3 (16.7)	6 (26.1)
**Provider providing comfort**			
Reassures the adolescent by clarifying his concerns/doubts	40 (90.9)	18 (100.0)	22 (95.7)
Tries to make the adolescent feel comfortable during the session	40 (90.9)	18 (100.0)	22 (95.7)
**Provider gives attention and respect**			
Shows/seems interested in what the adolescent has to say	35 (79.5)	14 (77.8)	21 (91.3)
Shows respect for the opinion and decisions of the adolescent	30 (68.2)	12 (66.7)	18 (78.3)
Uses body language that communicates paying full attention to the adolescent	40 (90.9)	18 (100.0)	22 (95.7)
Treats the adolescent with respect during the entire counseling session	44 (100.0)	18 (100.0)	23 (100.0)

Abbreviations: HIV, human immunodeficiency virus; VMMC, voluntary medical male circumcision.

^a^Analysis of observations by age category (10–14 vs 15–19 years) excluded observations where age was not indicated (n = 3).

One area of inconsistency in the counseling observations involved postprocedure care explanations. Adolescents were often not told how to evaluate for complications (eg, infection or bleeding; observed in only 65.9% overall) or what to do if complications arise (observed in 75.0% overall). In observed sessions, younger adolescents were provided the following content less often than older adolescents: the importance of abstaining from sex and masturbation for at least 6 weeks after the procedure (explained to 27.8% and 22.2%, respectively, in the younger group versus 56.5% and 47.8% in the older group); the need to use condoms even after receiving VMMC (38.9% vs 65.2%); condom demonstrations (0% vs 21.7%); and the need to reduce the number of sexual partners after VMMC (5.6% vs 52.2%).

Finally, regarding the tone of the counseling session, observers noted that in a majority of the sessions, providers showed interest in what the adolescent had to say (77.8% for those aged 10–14 and 91.3% for those aged 15–19 years), showed respect for the opinions and decisions of the adolescent (66.7% and 78.3%, respectively), and used body language that showed the counselor was paying full attention to the adolescent (90.9% overall). However, few providers told the youngest boys that they were going to ask personal questions during the session. Furthermore, providers infrequently assured adolescents of the confidentiality of the information they provided (assurance given to 11.1% of younger and 30.4% of the older adolescents).

## DISCUSSION

Male adolescents noted that although there was sufficient mention of VMMC benefits (including partial HIV protection) during counseling, little attention was paid to the procedure’s risks, including how to identify complications, what to do if they arise, and why avoiding sex and masturbation could prevent complications. The counseling content reported by adolescents revealed that older age, having ever had a sexual experience, and the presence of a postprocedure counseling session were factors associated with delivery of a more complete package. Notably, receiving group-based, less-individualized counseling was associated with reported lower levels of having received counseling content items, regardless of age group. Overall, older adolescents reported receiving more items from the recommended minimum package than younger adolescents. The self-reported findings were confirmed in the counseling observations, which revealed that condom demonstrations occurred less frequently for younger than for older adolescents. These deviations from the standard of care outlined by WHO and PEPFAR guidance [6, 7, 13], with opportunities missed for comprehensive wound care and prevention counseling, are concerning.

The current study’s findings are consistent with an observational study of VMMC counseling in Tanzania, which found that adolescents (aged 15–19 years) received more comprehensive information in individual counseling sessions than they did in group education when mixed with adults (aged ≥20 years) [14]. Studies of adult VMMC patients have found that counseling during VMMC service provision is indeed in need of improvement as well [15]; however, studies assessing adults’ report of counseling content suggest that men are receiving more complete messages regarding abstaining from sex after the procedure and the importance of consistent condom use despite VMMC status [16].

One possible way to improve and standardize the counseling content during VMMC service provision would be to provide VMMC counselors and providers with a checklist of the points they need to address in each and every counseling session, including those with adolescents. Surgical safety checklists have been shown to reduce adverse events such as life-threatening infections [17] and are recommended by WHO to increase communication and teamwork in surgery [18]. Consistent use of a checklist in VMMC counseling, with quality assurance and supervision ensured throughout, could potentially increase positive counseling outcomes. In study in Uganda, for instance, the quality of VMMC provision among adults, including patient counseling, improved dramatically after implementation of a quality assessment tool used by VMMC site management [19]. Such checklists and management tools could help counselors ascertain adolescent sexual activity, better tailor the counseling delivery appropriately, and provide complete and consistent information in every counseling session.

The rapid scale-up of VMMC has implications for preventing and monitoring adverse events, ensuring infection control, and providing appropriate postoperative counseling [20]. This study demonstrates that further work is needed to improve overall counseling quality, particularly for adolescent populations, and ensure the youngest boys are receiving the best possible standard of care and comprehensive prevention. Although WHO and PEPFAR provide recommendations on what should be covered during the VMMC service, counseling would be strengthened by specific training for counselors on delivering consistent messages in an age-appropriate manner. This may be particularly important if the counselor has biases concerning what he or she believes a young male adolescent needs to know, given his age and/or sexual experience [8, 21]. For instance, whereas all adolescents should receive comprehensive prevention messages, younger adolescents may require less technical descriptions to understand the content.

There are limitations to this study. Data from adolescents were self-reported and required recall up to 10 days after what might have been an anxiety-provoking experience. In addition, there may be selection bias, because only adolescents who completed their postprocedure interview were included in this analysis. However, a sensitivity analysis using multiple imputation to account for adolescents lost to follow-up found nearly identical results. A strength of this study was the use of mixed methods of data collection. Much of the counseling content adolescents received and retained was corroborated in direct observations of the counseling sessions. However, although we had a large sample of adolescents from a variety of settings, our sample size for direct counseling observations was limited.

Moving toward age-equitable comprehensive prevention for adolescent VMMC requires standardizing VMMC procedures as well as the counseling content. Any patient, regardless of age, deserves equal access to knowledge regarding the risks and benefits of VMMC and other HIV preventive measures. VMMC constitutes a tremendous opportunity to provide young male adolescents further counseling on HIV risks and prevention measures, counseling that can increase their self-efficacy and self-care and contribute to greater reductions in HIV acquisition and transmission.

## Supplementary Data

Supplementary materials are available at *Clinical Infectious Diseases* online. Consisting of data provided by the authors to benefit the reader, the posted materials are not copyedited and are the sole responsibility of the authors, so questions or comments should be addressed to the corresponding author.

## Supplementary Material

Kaufman_Content_SupplementalMaterial_ZP

87874_Supplemental_tables

## Notes

***Acknowledgments.*** We are grateful to the adolescent participants in this study for sharing their perspectives and experiences. We also thank the VMMC facility managers, community mobilizers, and providers for their support. We acknowledge the contributions of the Technical Advisory Group for the adolescent VMMC assessment and their guidance throughout the study. We are privileged to work with this group of dedicated professionals from PEPFAR, Centers for Disease Control and Prevention, the US Department of Defense, WHO, the United Nations Children’s Fund, and the Bill & Melinda Gates Foundation. Thank you to the Human Sciences Research Council in South Africa, CSK Research Solutions in Tanzania, PSI/Zimbabwe, and the Centre for Sexual Health & HIV/AIDS Research in Zimbabwe for their assistance with data collection. We also appreciate the assistance of Meaghen Murphy with copyediting and Maria Elena Figueroa with project start-up.

***Financial support.*** This work was supported by the United States Agency for International Development with PEPFAR funding (cooperative agreement AID-OAA-A-12-00058) to the Johns Hopkins Center for Communication Programs and cofunded by the UK Department of International Development through the Integrated Support Program in Zimbabwe.

***Supplement sponsorship.*** This article appears as part of the supplement “Adolescent Voluntary Medical Male Circumcision: Vital Intervention Yet Improvements Needed,” sponsored by Johns Hopkins University.

***Potential conflicts of interest.*** All authors: No reported conflicts. All authors have submitted the ICMJE Form for Disclosure of Potential Conflicts of Interest. Conflicts that the editors consider relevant to the content of the manuscript have been disclosed.
