# Supplementary material for: Counseling Received by Adolescents Undergoing Voluntary Medical Male Circumcision: Moving Toward Age-Equitable Comprehensive Human Immunodeficiency Virus Prevention Measures
Source: Clin Infect Dis. 2018 Apr 3;66(Suppl 3):S213–20. doi: 10.1093/cid/cix952 (PMC5889033; doi:10.1093/cid/cix952)
Supplement: Kaufman_Content_SupplementalMaterial_ZP [file cix952_suppl_kaufman_content_supplementalmaterial_zp.docx]

**SUPPLEMENTAL INFORMATION**

**Supplementary Table S1.** Sensitivity analysis of key findings using multiple imputation to account for potential selection bias (N=1526).

| **Multiple Imputation Results** | | | |
| --- | --- | --- | --- |
|  | **Age Group, y** | **PR (95% CI)** | **aPR (95% CI)** |
| Complete VMMC subindex | 10-14  15-19 | Ref.  **1.15 (1.03-1.29)** | Ref.  1.12 (0.99-1.26) |
| Complete HIV test promotion subindex | 10-14  15-19 | Ref.  **1.54 (1.20-1.97)** | Ref.  **1.51 (1.15-1.99)** |
| Complete condom subindex | 10-14  15-19 | Ref.  **2.74 (1.44-5.21)** | Ref.  **2.25 (1.26-4.00)** |
| Complete consent/assent (proxy) | 10-14  15-19 | Ref.  **1.17 (1.04-1.33)** | Ref.  1.14 (1.00-1.29) |
| Receipt of HIV testing | 10-14  15-19 | Ref.  1.02 (0.96-1.08) | Ref.  1.01 (0.95-1.07) |

Multiple imputation was conducted using chained equations (m=20). Facility area and were included in the imputation model to improve stability. The outcomes shown were a complete sub-index (VMMC subindex: 2/2; HIV test promotion subindex: 2/2; condom subindex: 2/2; consent/assent proxy 2/2) and receipt of HIV testing. Prevalence ratios (PR) were calculated by modified Poisson regression models with GEE and robust variance estimators to account for clustering of responses at the facility level. Estimates in bold have a *P* value < 0.05. Multivariable models for each sub-index included adjustment for country, pre-procedure counseling mode, ever having had a sexual experience and receipt of a postprocedure counseling session.
