# Supplementary material for: Counseling Received by Adolescents Undergoing Voluntary Medical Male Circumcision: Moving Toward Age-Equitable Comprehensive Human Immunodeficiency Virus Prevention Measures
Source: Clin Infect Dis. 2018 Apr 3;66(Suppl 3):S213–20. doi: 10.1093/cid/cix952 (PMC5889033; doi:10.1093/cid/cix952)
Supplement: 87874_Supplemental_tables [file cix952_suppl_87874_supplemental_tables.pdf]

Supplementary Table S2. Uptake of HIV testing by age group and country.

Age Group, years	% (n/N)	PR (95% CI)	aPR (95% CI)
South Africa			
10-14	94.7% (177/187)	Ref.	Ref.
15-19	98.2% (110/112)	1.04 (0.99-1.09)	1.00 (0.99-1.02)
Tanzania			
10-14	81.4% (336/413)	Ref.	Ref.
15-19	87.1% (74/85)	1.00 (0.80-1.25)	0.95 (0.82-1.10)
Zimbabwe			
10-14	94.5% (223/236)	Ref.	Ref.
15-19	95.0% (247/260)	1.00 (0.97-1.04)	0.99 (0.97-1.01)

The outcome was uptake of HIV testing. Prevalence ratios (PR) were calculated by modified Poisson regression models with generalized estimating equations and robust variance estimators to account for clustering of responses at the facility level. Each country-stratified multivariable model included adjustment for facility setting. All P values were >0.05.

Supplementary Table S3. Sensitivity analysis of key findings using multiple imputation to account for potential selection bias.

Multiple Imputation Results				
	**Age Group, years**	**PR (95% CI)**	**aPR$_1$ (95% CI)**	**aPR$_2$ (95% CI)**
VMMC sub-index	10-14	Ref.	Ref.	Ref.
	15-19	**1.15 (1.02-1.30)**	**1.15 (1.01-1.30)**	1.06 (0.95-1.19)
HIV sub-index	10-14	Ref.	Ref.	Ref.
	15-19	**1.50 (1.19-1.91)**	**1.52 (1.20-1.92)**	**1.54 (1.17-2.01)**
Condom sub-index	10-14	Ref.	Ref.	Ref.
	15-19	**2.73 (1.41-5.29)**	**2.76 (1.37-5.56)**	**2.43 (1.28-4.63)**
Consent (Proxy)	10-14	Ref.	Ref.	Ref.
	15-19	1.09 (0.97-1.23)	1.09 (0.97-1.23)	1.09 (0.95-1.25)
HIV test uptake	10-14	Ref.	Ref.	Ref.
	15-19	1.02 (0.97-1.07)	1.02 (0.97-1.07)	1.02 (0.97-1.07)

The outcomes were a complete sub-index (VMMC sub-index: 2/2; HIV sub-index: 3/3; Condom sub-index: 2/2; Consent Proxy 1/1) and HIV testing uptake. Prevalence ratios (PR) were calculated by modified Poisson regression models with generalized estimating equations and robust variance estimators to account for clustering of responses at the facility level. Estimates in bold have a *P* value < 0.05.

Multiple imputation was conducted using chained equations (m=20).

[1] Multivariable models for each sub-index included adjustment for country.

[2] Final multivariable models for each sub-index included adjustment for country, pre-procedure counseling mode, ever had a sexual encounter, and receipt of post-procedure counseling.
